# Trauma Communications Center Coordinated Severity-Based Stroke Triage: Protocol of a Hybrid Type 1 Effectiveness-Implementation Study

**DOI:** 10.3389/fneur.2021.788273

**Published:** 2021-12-06

**Authors:** Toby I. Gropen, Nataliya V. Ivankova, Mark Beasley, Erik P. Hess, Brian Mittman, Melissa Gazi, Michael Minor, William Crawford, Alice B. Floyd, Gary L. Varner, Michael J. Lyerly, Camella C. Shoemaker, Jackie Owens, Kent Wilson, Jamie Gray, Shaila Kamal

**Affiliations:** ^1^Division of Cerebrovascular Disease, The University of Alabama at Birmingham, Birmingham, AL, United States; ^2^The University of Alabama at Birmingham, Birmingham, AL, United States; ^3^Vanderbilt University Medical Center, Nashville, TN, United States; ^4^Kaiser Permanente Southern California, Pasadena, CA, United States; ^5^The Office of Emergency Medical Services, Alabama Department of Public Health, Montgomery, AL, United States; ^6^The Office of Emergency Medical Services, Alabama Department of Public Health, Prattville, AL, United States; ^7^Mobile Infirmary Medical Center, Mobile, AL, United States

**Keywords:** large vessel occlusion, mechanical thrombectomy, prehospital care, emergency medical service, trauma communications centers, mixed methods research, implementation science, delivery of health care

## Abstract

**Background:** Mechanical thrombectomy (MT) can improve the outcomes of patients with large vessel occlusion (LVO), but a minority of patients with LVO are treated and there are disparities in timely access to MT. In part, this is because in most regions, including Alabama, the emergency medical service (EMS) transports all patients with suspected stroke, regardless of severity, to the nearest stroke center. Consequently, patients with LVO may experience delayed arrival at stroke centers with MT capability and worse outcomes. Alabama's trauma communications center (TCC) coordinates EMS transport of trauma patients by trauma severity and regional hospital capability. Our aims are to develop a severity-based stroke triage (SBST) care model based on Alabama's trauma system, compare the effectiveness of this care pathway to current stroke triage in Alabama for improving broad, equitable, and timely access to MT, and explore stakeholder perceptions of the intervention's feasibility, appropriateness, and acceptability.

**Methods:** This is a hybrid type 1 effectiveness-implementation study with a multi-phase mixed methods sequential design and an embedded observational stepped wedge cluster trial. We will extend TCC guided stroke severity assessment to all EMS regions in Alabama; conduct stakeholder interviews and focus groups to aid in development of region and hospital specific prehospital and inter-facility stroke triage plans for patients with suspected LVO; implement a phased rollout of TCC Coordinated SBST across Alabama's six EMS regions; and conduct stakeholder surveys and interviews to assess context-specific perceptions of the intervention. The primary outcome is the change in proportion of prehospital stroke system patients with suspected LVO who are treated with MT before and after implementation of TCC Coordinated SBST. Secondary outcomes include change in broad public health impact before and after implementation and stakeholder perceptions of the intervention's feasibility, appropriateness, and acceptability using a mixed methods approach. With 1200 to 1300 total observations over 36 months, we have 80% power to detect a 15% improvement in the primary endpoint.

**Discussion:** This project, if successful, can demonstrate how the trauma system infrastructure can serve as the basis for a more integrated and effective system of emergency stroke care.

## Introduction

Just as trauma systems have proven ability to save lives of the most severely injured patients, we should have a stroke system able to provide care to patients with the most severe strokes. The most severe type of acute ischemic stroke is due to proximal large vessel occlusion (LVO). In one systematic review, LVO strokes represented ~40% of all acutely presenting ischemic strokes but accounted for 62% of post-stroke disabilities and 96% of post-stroke mortality (1). Mechanical thrombectomy (MT) offers an extraordinary potential to improve the outcome of patients with LVO (2). Unfortunately, in part because MT is available only at advanced stroke centers with MT capability (MTC), only a minority of patients with LVO are treated with MT (3). Moreover, there are racial, socioeconomic, and urban-rural disparities in access to MT (4, 5).

While the development of brief stroke severity scales and multiple stroke center designations (analogous to different levels of trauma centers) are important pieces of the acute stroke system, there are still opportunities to improve regionally organized and integrated acute stroke care (6). The US stroke system has been described as a “pseudo-regionalized,” uncoordinated system lacking systematic oversight and control (7). In most acute stroke systems of care, including Alabama, stroke patients are triaged to the nearest stroke center, regardless of stroke severity. This is a critical problem because inter-facility transfer of LVO patients from a facility that does not offer thrombectomy to one that does often delays or precludes treatment and results in worse outcomes (8). Based on the limited available data, the American Heart Association Mission: Lifeline Stroke Committee developed a consensus algorithm for severity-based stroke triage (SBST) in 2018, updated in 2020 (9). Per the algorithm, our acute stroke care system should prioritize emergency medical service (EMS) triage of patients with suspected LVO to a MTC unless transport time to a MTC would disqualify treatment with tissue plasminogen activator (tPA). While the concept is reasonable, there is still no clinical trial data supporting the benefits of regional implementation of SBST compared to triage of all stroke patients to the nearest stroke center.

A critical barrier to improving broad, equitable, and timely access of patients with LVO to proven therapy and better health outcomes is the lack of an integrated acute stroke care system. Fargen et al. (7) observed that patients with severe stroke and trauma are similar in several respects: they are critically ill, require specialized often multi-disciplinary care, and have a finite time window to receive life-saving therapies. We agree that the next step is the development of well-integrated regional systems of stroke care using the highly evidenced US regional trauma systems as a blueprint. A trauma system may be defined as an “organized, coordinated effort in a defined geographic area that delivers the full range of care to all injured patients and is integrated with the local public health system” (10). Trauma systems have proven ability to save lives (11) and improve functional outcomes (12). An important factor in successful trauma systems has been integration of trauma care services into a regionalized system including the ability to transport appropriate patients from the scene directly to tertiary centers (13). Trauma communications centers (TCCs) are a critical component of larger trauma systems, assisting first responders in the field with the coordination, communication, information, and in some cases determination of where and how injured patients are transported (14).

This project proposes to leverage existing trauma system infrastructure as the basis for a more integrated and effective system of emergency stroke care in Alabama. In our preliminary study, we established the feasibility of improving prehospital LVO recognition by establishing unique real-time TCC directed performance of the Emergency Medical Stroke Assessment (EMSA) in the Birmingham region (15). The intervention was designed to address three barriers to prehospital stroke care, including limited stroke-specific training of EMS, infrequent exposure of individual EMS providers to stroke, and limited feedback on performance (16, 17), by focusing training, experience, and feedback on a small number of Alabama TCC paramedic communicators. The Alabama Department of Public Health (ADPH) has initiated a 5-year statewide quality improvement program of TCC Coordinated SBST which aims to transform the acute stroke care system by coordinating prehospital and inter-facility emergency stroke care. This provides a “natural experiment” allowing assessment of both the public health impact and “how and why” of implementation of an innovative acute stroke care model.

## Study Aims

Our aims are to (1) Develop a stroke triage care model, trauma communications center (TCC) coordinated severity-based stroke triage (SBST), designed to coordinate prehospital and inter-facility emergency stroke care; (2) Investigate the comparative effectiveness of this care pathway for improving broad, equitable, and timely access to MT; and (3) Explore stakeholder perceptions of the intervention's feasibility, appropriateness, and acceptability using a mixed methods approach.

## Methods and Analysis

### Alabama Stroke System

**Figure 1:**
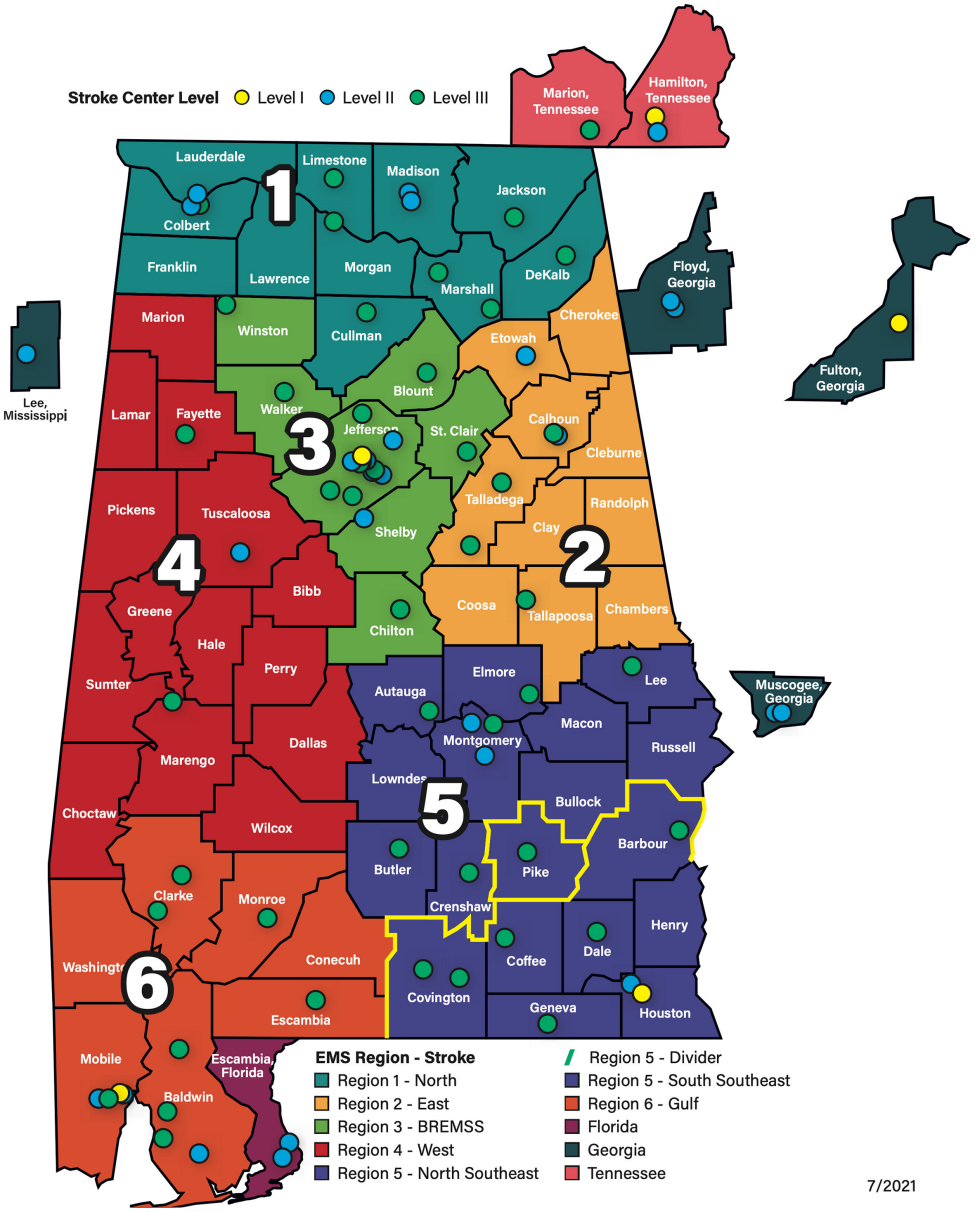
Alabama EMS Regions and Stroke Centers. The Alabama stroke system has 80 stroke centers. Level III centers (green dots) are acute stroke-ready hospitals, level II centers (blue dots) are primary stroke centers, and Level I centers (yellow dots) are comprehensive stroke centers. See text for details regarding primary stroke centers that are currently thrombectomy-capable. Map Source: ADPH.

The statewide stroke emergency care system is managed by the ADPH Office of Emergency Medical Services (OEMS). Alabama counties are assigned to one of six EMS Regions shown in Figure 1. The OEMS oversees EMS Regional Agencies that provide initial contact for EMS concerns, provide effective communication between communities and the OEMS, assist EMS with rules compliance, provide local credentialing, and function as a clearing house for EMS education. In each region, established EMS Regional Directors and Advisory Councils are responsible for direct oversight and management of its specific regional stroke system. In 2017, the ADPH OEMS developed a statewide stroke system with three levels of stroke centers. Currently, the Alabama stroke system has 80 stroke centers, with 14, 7, 17, 3, 17, and 11 centers in EMS regions 1–6, respectively, and 11 out of state centers as shown in Figure 1. Level III centers (green dots) are acute stroke-ready hospitals, level II centers (blue dots) are primary stroke centers. There are three level I comprehensive stroke centers (yellow dots) in Alabama, the University of Alabama at Birmingham (UAB) in region 3; Southeast Health Medical Center, Dothan, in region 5; and University of South Alabama, Mobile, in region 6. Out of state comprehensive stroke centers include Erlanger Hospital, Chattanooga TN and Grady Memorial Hospital, Atlanta GA.

At the heart of the system is a single Emergency Communications Center for the State, known as the Alabama Trauma Communications Center (hereafter referred to as TCC). Funding from member hospitals and an ADPH grant supported the development and implementation of the TCC in 2007 (14). The TCC is operated by the Birmingham Regional Emergency Medical Services System (Alabama EMS Region 3), which is overseen by the ADPH OEMS. EMS providers across the State enter all patients with suspected stroke into the Alabama Stroke System by calling the TCC. The 19 paramedic communicators of the TCC maintain the current status of hospitals and resources across the state. The TCC is not involved with initial emergency medical dispatch, but rather assists EMS by routing the patient to the nearest stroke system hospital depending on hospital-resource availability and notifying the receiving hospital. Hospitals continually update their stroke patient resource availability. All interactions between TCC and EMS are recorded, facilitating quality improvement. Patients entered in the stroke system are assigned a TCC number unique for each stroke system patient entry which will enable tracking patients who undergo inter-facility transfer. EMS providers currently provide TCC with patient data including the Face, Arm, and Speech Test (18), time last known well, and level of responsiveness using the Alert, responds to Voice, responds to Pain and Unresponsive Scale (19). Additionally, stroke center coordinators currently provide feedback to TCC on stroke system patients, including whether stroke was confirmed, stroke diagnosis, and whether the patient received tPA treatment. Prehospital as well as hospital data on all stroke system patients is entered into the ADPH's custom-built secure electronic data capture system (LifeTrac) by the TCC paramedic communicators.

### Study Organization

On the basis of the MT clinical trial data, the published American Heart Association Mission: Lifeline Stroke SBST Algorithm for EMS (9), a recent policy statement on stroke systems of care (20), and the results of our preliminary study (15), the ADPH OEMS is implementing TCC Coordinated SBST across Alabama's six EMS regions. This is an observational study of the public health impact and implementation of this quality improvement initiative. While the stroke system change is being carried out under the auspices of the OEMS, the OEMS has been advised by the Stroke System Subcommittee of the Statewide Trauma and Health Systems Advisory Council. The Stroke System Subcommittee draws on stroke expertise from across the State. As necessary components of this stroke system change, the ADPH is adding a designation for thrombectomy-capable primary stroke centers. Currently, thrombectomy-capable primary stroke centers include Huntsville Hospital in region 1; Brookwood Baptist Medical Center, Homewood, in region 3; Baptist Medical Center South, Montgomery, in region 5; and Mobile Infirmary in region 6. In addition, the ADPH is carrying out EMS training in the EMSA across the state and expanding LifeTrac data collection to capture statewide stroke system data including prehospital EMSA items (gaze, facial droop, arm drift, leg drift, naming, and repetition) and hospital data including whether an LVO was confirmed, LVO location, and whether the patient received mechanical thrombectomy. The State Medical Director of the OEMS (WC) will provide medical oversite for the OEMS as implementation goes statewide and will monitor for patient care and/or quality issues, EMS issues and hospital issues, and will provide updates to the Statewide Trauma and Health Systems Advisory Council.

### Design of the Stepped Wedge Cluster Trial

**Figure 2:**
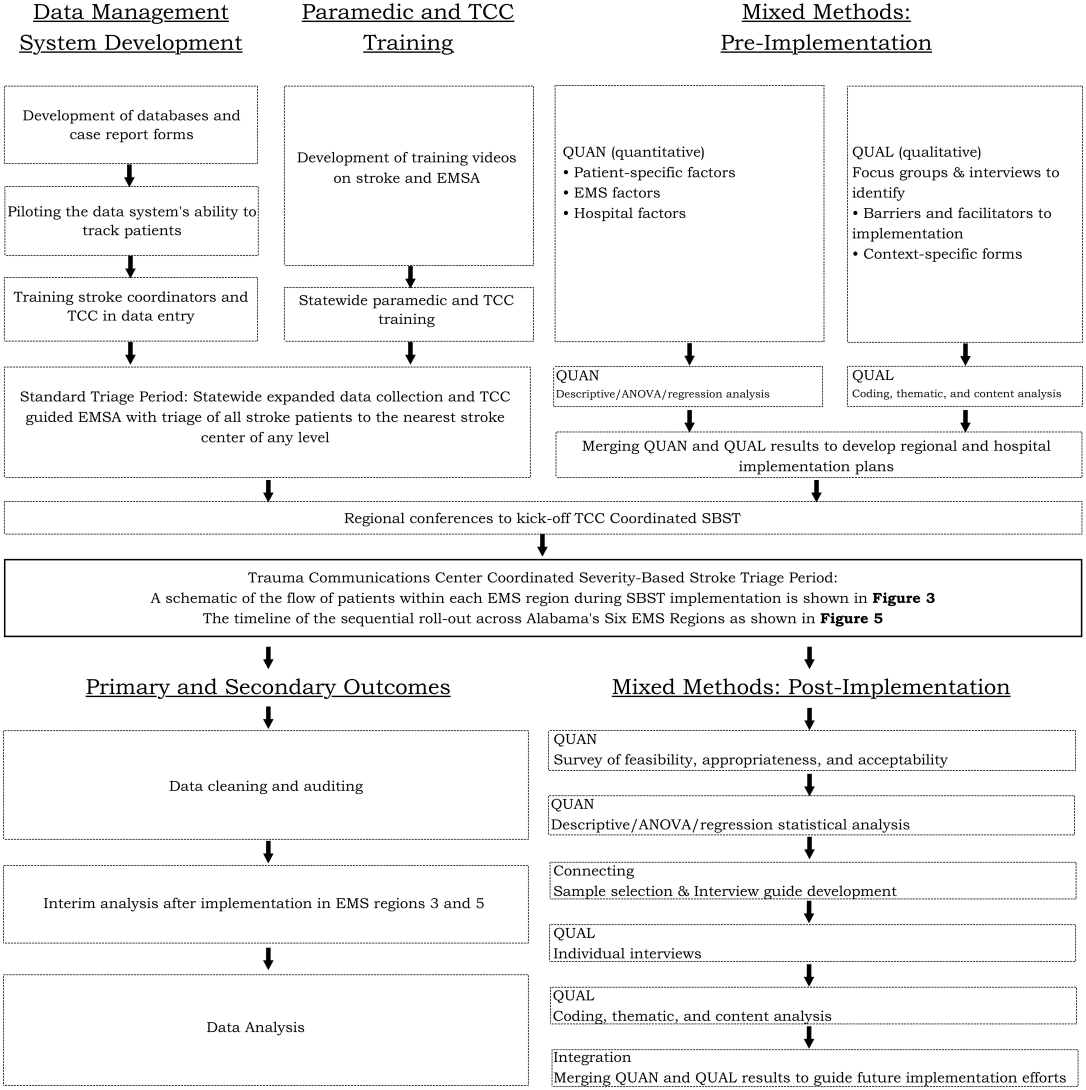
Study flowchart of hybrid type 1 effectiveness-implementation study with a multi-phase mixed methods sequential design and an embedded observational stepped wedge cluster trial.

This is a hybrid type 1 effectiveness-implementation study with a multi-phase mixed methods sequential design and an embedded observational stepped wedge cluster trial. For an overview of how the study elements fit together see the study flowchart (Figure 2). The change in health care policy by the ADPH allows a natural experiment. Given available resources and the complexity of the intervention, the ADPH has planned a phased rollout, lending itself to analysis as a stepped wedge cluster trial with each EMS region serving as a cluster (21, 22). A stepped wedge cluster trial is appropriate given the existing evidence in support of stroke system change, the logistical need for sequential rollout, and the plan for the entire state (i.e., all clusters) to eventually implement the system change. This design will facilitate modeling the impact of time on the effectiveness of TCC Coordinated SBST. This study will broadly and equitably target a population of stroke patients of all ages, sexes, races, and ethnicities. A strength of this study is that at least 50% of subjects will be women and over one-third of the enrolled patients will be African Americans based on census data. As the study involves rural and urban regions and stroke centers of all levels across the State, we will gain information regarding the impact on resources (e.g., EMS response times for non-stroke conditions), benefits, and potential risks (e.g., delay to tPA treatment and other unintended consequences) of implementation across a wide range of service delivery contexts. Finally, because the trial is embedded within expanded LifeTrac data collection on all stroke system patients as well as system level data captured by ADPH, we will be able to evaluate its public health impact.

### Mixed Methods Multi-Phase Design

A recent prehospital stroke system of care consensus conference advised collaboration of regional stakeholders to create an optimally adapted prehospital stroke system (23). A mixed methods methodological approach which aims to integrate rigorous quantitative and qualitative methods within a study will be the most effective way to understand varied and multi-level stakeholder perceptions and optimize implementation of this complex health intervention across different service contexts (24). We will use a multi-phase QUAL+QUAN → QUAN → QUAL mixed methods design to assess stakeholders' views about the implementation process capitalizing on the advantages of integrating quantitative and qualitative methods using surveys and quantitative measures, focus groups and individual semi-structured interviews (24–27). We will use quantitative and qualitative methods to aid in the development of context specific forms of the model prior to implementation (28–30). Quantitative data will include patient-specific factors (EMSA score, Alert, responds to Voice, responds to Pain and Unresponsive scale, time last known well, potential tPA eligibility, vital signs), EMS factors (EMS air and ground resources, estimated travel times to nearest available non-MTC and MTC), and hospital factors (stroke center level, imaging, and treatment capabilities, location). Qualitative data consisting of focus groups and interviews with stakeholders will be critical for development of regional triage plans and stroke center protocols. An example Focus Group Guide for the TCC paramedic communicators is available (Supplementary Material 1). After implementation, we will survey stakeholders to assess perceptions of feasibility, appropriateness, and acceptability of the intervention and employ follow-up qualitative interviews with purposefully selected individuals to understand context-specific barriers and facilitators more fully to intervention adoption, implementation, maintenance, and spread. Systematic integration and triangulation of quantitative and qualitative data will assure more valid conclusions (31).

### Sampling Strategy

Understanding the perspectives of purposefully selected (32) key stakeholders will facilitate implementation and evaluation of an intervention that is complex, with care provided by multiple types of providers with different roles, at multiple locations, at different stages of hyperacute stroke care. Key stakeholders include TCC paramedic communicators, EMS regional directors and advisory councils, EMS providers, stroke center coordinators, and stroke center directors. We also plan inclusion of stakeholders that provide care and/or oversight in a variety of service contexts with variable resources including rural and urban regions and stroke centers of various levels.

### Patient Population—Inclusion and Exclusion Criteria

**Figure 3:**
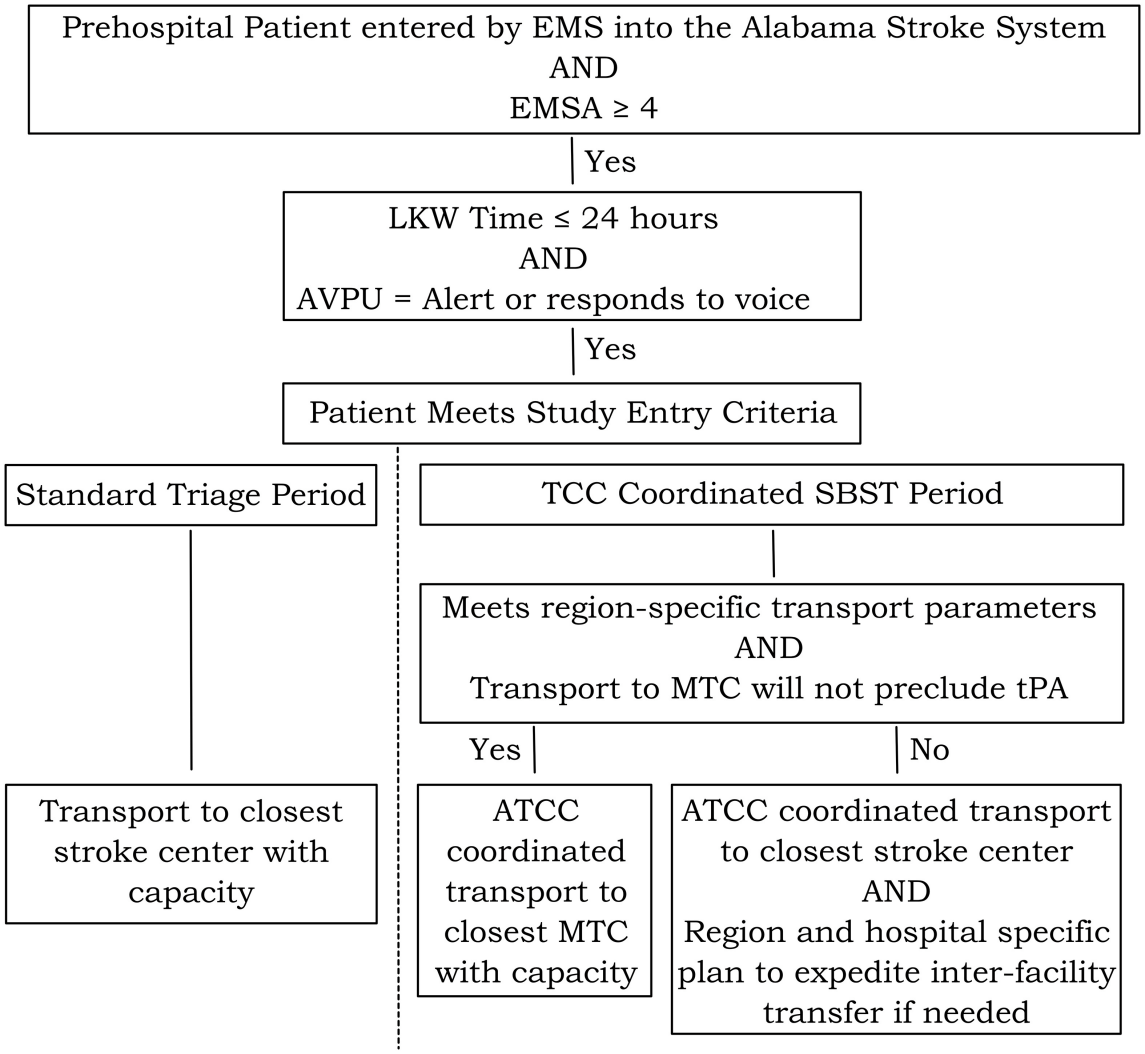
Flow of patients during study. The study will include prehospital patients entered into the stroke system by EMS with an EMSA ≥ 4. Patients with a time last known well (LKW) > 24 h or those who respond only to pain or who are unresponsive will be excluded. During the standard triage period, patients meeting study entry criteria will be transported to the closest stroke center with capacity. During TCC Coordinated SBST, patients will be routed by TCC directly to a MTC (including comprehensive stroke centers and thrombectomy-capable primary stroke centers) if additional transport time complies with region-specific transport time limits and will not preclude use of tPA. Otherwise, TCC will coordinate transport to the closest stroke center of any level and initiate a region and hospital specific plan to expedite inter-facility transfer to a MTC for appropriate patients.

The study will include patients entered in the Alabama stroke system by EMS with suspected LVO based on an EMSA ≥ 4 with one point scored for each abnormal response on the 6-item scale. The study will exclude patients with stroke onset while hospitalized, a time last known well ≥24 h prior to initial EMS contact, and patients who only respond to pain or who are unresponsive based on the Alert, responds to Voice, responds to Pain and Unresponsive Scale (19). The flow of patients during the study is shown in Figure 3.

### Randomization

Not applicable as this is a stepped wedge trial with the order of regional implementation determined by the ADPH.

### Intervention

The implementation of TCC Coordinated SBST will be carried out under the auspices of the ADPH with the overarching goal to address current stroke system challenges. The *sine qua non* of this initiative is coordination of SBST by the TCC. Because this is a statewide initiative and the TCC serves the entire state, some elements of the intervention such as the use of the EMSA will be standardized across the state, as shown in Table 1. At the level of EMS regions and stroke centers, however, the specific strategies used to achieve TCC Coordinated SBST will be tailored to the specific context. Context-specific adaptation will depend on local resources and practice. For example, longer travel times in rural compared to urban regions might prompt longer additional transport time limits in rural regions to route a patient past a non-MTC to a MTC. Another example is that non-MTCs will have variable imaging and telestroke capability and hence site-specific code stroke protocols. Acute stroke system challenges, TCC Coordinated SBST core functions, and context-specific forms that might be used to achieve the core functions are shown in Table 2. Implementation plans are as follows:

(1) EMS Training in the EMSA: As previously noted, our model was designed to address barriers to prehospital stroke care (16, 17), by focusing training, experience, and feedback on the TCC paramedic communicators. However, we applied what we have learned from our preliminary study to enhance our training program for EMS providers in the field (15). For example, we included explicit strategies in the EMSA to enable determination of abnormal horizontal gaze or lateralized weakness of the face, arm, or leg in confused or aphasic patients. The training program was also informed by prior qualitative studies on paramedic decision-making for high acuity patients (mostly trauma) that have found a reliance on the paramedic's initial “gut” impression (33) and a study that found that the diversity of stroke presentations was a barrier to acute stroke recognition by paramedics (34). We addressed these issues by providing “snapshot” figures representing left and right middle cerebral artery syndromes in addition to training on individual scale items to aid EMS providers who favor a gestalt approach to diagnosis. We created two training videos for presentation at regional zoom kick-off events including EMS agencies and hospitals. A 24 min video, “Severity-Based Stroke Triage: Key Concepts,” covers the State's plan for roll-out of severity-based stroke triage, stroke vs. stroke mimics, large vessel occlusion, the EMSA, treatment options for acute ischemic stroke, and the concept of time last known well (35). The other video is a 9 min demonstration of the “Emergency Medical Stroke Assessment” by paramedics (36). After the regional kick-off events, paramedics across the state were required to view the training videos and complete a 20 question post-training examination. The EMS training videos and examination take <90 min to complete.
(2) EMS stroke severity assessment and prenotification: After statewide EMSA training is complete, TCC Guided EMSA with item-specific prenotification and focused feedback will be expanded to all EMS regions in Alabama. Key elements from the preliminary study that have been incorporated into the current protocol include TCC guidance of EMS performance of the EMSA when needed, item-specific prenotification by TCC rather than a summary score, and ongoing review of recorded interactions between TCC and EMS with feedback to TCC to enhance their guidance skills (15). During the standard triage data collection period in each EMS region, all stroke patients (including those with suspected LVO based on an EMSA ≥ 4) will undergo triage to the nearest stroke center with capacity of any level (Figure 3). Additional training for TCC will be employed as needed to optimize performance of the TCC Guided EMSA.
(3) Prehospital Severity-Based Stroke Triage: Plans will be developed during the standard triage period in each region considering EMS and hospital resources, levels and numbers of stroke centers, travel times by ground and air in each region, and data from focus groups and interviews. A schematic of the flow of patients within each EMS region during SBST implementation is shown in Figure 3. During implementation, patients entered into the stroke system by EMS who have an EMSA ≥ 4 with a time last known well ≤ 24 h who are alert or respond to voice will be triaged as follows: If direct transport to a MTC (including comprehensive stroke centers and thrombectomy-capable primary stroke centers) complies with region-specific transport time limits and will not preclude use of tPA, TCC will coordinate pre-notification and air or ground transport to a MTC. If direct transport time to a MTC is greater than region-specific limits or transport to a MTC will preclude use of tPA, TCC will coordinate pre-notification and air or ground transport to the closest available stroke center of any level and initiate a region and hospital specific plan to expedite inter-facility transfer to a MTC.
(4) Emergency Department Severity-Based Stroke Triage: The implementation strategy includes an educational conference to be held in each region to review regional implementation plans, foster engagement of stroke center personnel and EMS, provide a “toolkit” of resources to streamline care, and train stroke coordinators throughout Alabama. The grant supports reimbursement for stroke coordinators who successfully pass the Stroke Certified Registered Nurse examination offered American Board of Neuroscience Nursing (37). The plan includes interviews and focus groups with stroke center directors and coordinators, on-site evaluation if possible, protocol development, and training on stroke treatment options and the EMSA. We will encourage EMSA performance systematically as part of code strokes on patient arrival. Depending on available resources, context specific forms might include multimodal computerized tomography (CT) including noncontrast CT, CT angiography, and CT perfusion on code stroke patients with suspected LVO, telestroke or image sharing with MTCs, and transitioning from alteplase to tenecteplase to facilitate rapid treatment and transfer.
(5) Inter-facility Severity-Based Stroke Triage: Plans for secondary triage will be developed during the standard triage period and will depend on regional resources as well as data from focus groups and interviews. The plan includes TCC monitoring of the status of patients with suspected LVO who are initially transported to non-MTCs, modeled after the TCC Trauma system process. In this process, the TCC follows up with the ED every 20 min to update patient status and identify the best options for secondary triage in real-time. Context specific strategies might include holding the EMS unit involved with primary triage of patients with suspected LVO at the non-MTC until a transfer decision is made or tPA is initiated, modeled after the Alabama Trauma system “Rescue Stop.” Alternatively, TCC might proactively mobilize ground or air resources to achieve rapid patient transfer.

**Table 1:** Acute stroke system challenges and statewide TCC coordinated SBST elements.

**Stroke system components and challenges**	**Statewide TCC coordinated SBST elements**
**Emergency Medical System (EMS) provider training and stroke knowledge retention** • Limited EMS stroke training, experience, and feedback • Large workforce, frequent staff turnover • Stroke mimics and diversity of stroke presentations • Training emphasizes algorithmic rather than impressionistic approach	• In depth training of the 19 TCC paramedic communicators • Statewide paramedic training with two videos (see text) and a 20 question post-training examination • Incorporation of strategies to detect lateralized findings in confused or aphasic patients in the Emergency Medical Stroke Assessment (EMSA) cards and videos • Inclusion of “snapshot” figures to assist in rapid “gut” impression of Middle Cerebral Artery syndromes in videos
**EMS stroke severity assessment and prenotification** • Limited EMS medical control for stroke • Variable LVO scale use • Stroke diagnostic challenges • Variable pre-notification	• TCC guides EMS in EMSA performance as needed • TCC assists with evaluation of patients unable to follow commands and to detect neglect • Item-specific prenotification by TCC • Focused feedback to TCC based on review of recorded interactions between ATCC and EMS to enhance guidance skills

*TCC, Trauma Communications Center; SBST, Severity-Based Stroke Triage; LVO, Large Vessel Occlusion*.

**Table 2:** Acute stroke system challenges, TCC Coordinated SBST core functions, and examples of forms.

**Stroke system components and challenges**	**TCC coordinated SBST core function (required)**	**Examples of forms (strategies that are optional or may be tailored to achieve core functions)**
**Prehospital SBST** • Limited adoption of SBST • Limited data to support SBST	• Interviews with EMS Regional Directors to develop region/county specific SBST transport protocols • TCC assists in choice of destination and coordination of transport mode (ground vs. air)	• Longer transport limits in rural and suburban regions compared to urban locations • Modification of transport limits by resource availability • Air transport from rural locations when weather permits • Patient “hand-off” from one EMS unit to another to facilitate longer transports and keep units available in regions with limited EMS resources • Mobilization of back-up EMS units in regions with limited EMS resources when an out of region transport is initiated
**Emergency Department (ED) SBST** • Variable LVO scale use in the ED • Delays to cerebrovascular imaging • Delays to initiation of transfer process at non-MTCs • Delayed mobilization of team for inter-facility transfer • Limited transport resources, especially in rural areas	• Interviews with stroke center directors and coordinators to develop stroke center protocols • Regional symposia to review SBST plans, train coordinators, and provide “toolkit” to streamline ED stroke care • Active TCC monitoring in the ED every 20 min for suspected LVO patients prior to transfer • TCC assists in choice of MTC for transfer	• EMSA or another LVO scale included in code stroke on patient arrival • NIHSS included in code stroke on patient arrival • Combined CT/CTA on code stroke patients with suspected LVO • Combined CT/CTA/CTP on code stroke patients with suspected LVO • Telestroke consultation • Image sharing with MTC • Transition from alteplase to tenecteplase to facilitate rapid treatment and transfer • TCC proactively mobilizes transport team for secondary triage • Initial EMS unit remains until transfer decision is made (“Stroke Rescue Stop”) • Mobilization of neuro-interventional team once transfer decision is made • Transport to MTC ED or directly to angiography suite

*TCC, Trauma Communications Center; SBST, Severity-Based Stroke Triage; LVO, Large Vessel Occlusion; MTC, Mechanical Thrombectomy Center; CT, Computerized Tomography; CTA, CT Angiography; CTP, CT Perfusion*.

### Primary Outcome

The primary outcome is change in the proportion of prehospital stroke system patients with suspected LVO who are treated with mechanical thrombectomy before and after implementation of TCC Coordinated SBST. We hypothesize that compared to standard triage, TCC Coordinated SBST will be associated with a significant increase in the proportion of patients encountered by EMS with suspected LVO who are treated with MT.

### Secondary Outcomes

Consistent with the aims of implementation research, in addition to effectiveness, we seek to fully assess change in the broad public health impact before and after implementation of TCC Coordinated SBST using the RE-AIM (Reach, Effectiveness, Adoption, Implementation, and Maintenance) framework (AIM 2) (38, 39). The public health outcomes of TCC Coordinated SBST across RE-AIM dimensions are shown in Table 3. There may be unintended negative consequences to implementation of TCC Coordinated SBST, including delay in access to tPA and longer EMS response times. We address these concerns by explicitly tracking the impact of the intervention on these outcomes (see Table 3). Another concern is overloading MTCs with patients without LVO due to false positive screens and reducing capacity of MTCs to accept transfers of patients for MT. In part, we are hoping to mitigate against this by adding a designation in Alabama for thrombectomy-capable primary stroke centers. We will also track the proportion of LVO patients requiring inter-facility transfer, the timeliness of transfer, and rate of MT among all confirmed LVO strokes for patients entered into the Alabama Stroke System (see Table 3). As part of the stepped wedge trial, we will collect data on the rate of true positive and false positive LVO screens. Because statewide stroke system data collection will include whether an LVO was confirmed, LVO location, and whether the patient received MT, we will be able to determine the rate of true negative and false negative LVO screens, and accordingly, sensitivity and specificity of prehospital LVO screening.

**Table 3:** RE-AIM framework to evaluate the public health impact of TCC Coordinated SBST.

**RE-AIM dimension**	**Outcomes**	**Data source**
**Reach**	• Proportion of Alabama MT patients who undergo TCC Coordinated SBST	• ADPH RESCUE ePCR and LifeTrac database
	• Differential reach by race, ethnicity, sex, and population density	• ADPH RESCUE ePCR and LifeTrac database
**Effectiveness**	• Proportion and timeliness of MT for EMS-suspected LVO	• LifeTrac and ADPH REDCap databases
	• Proportion and timeliness of MT for all confirmed Stroke System LVO	• LifeTrac and ADPH REDCap databases
	• Proportion and timeliness of tPA treatment	• LifeTrac and ADPH REDCap databases
	• Proportion requiring inter-facility transfer and timeliness of transfer	• LifeTrac and ADPH REDCap databases
	• Differential effectiveness by race, ethnicity, sex, and population density	• LifeTrac and ADPH REDCap databases
	• Differential effectiveness for patients initially triaged to a non-MTC	• LifeTrac and ADPH REDCap databases
	• modified Rankin Scale 3 months after discharge	• ADPH REDCap databases
	• County/Regional EMS response times	• ADPH RESCUE ePCR
**Adoption**	• Proportion of Alabama EMS organizations that participate	• ADPH RESCUE ePCR and LifeTrac database
	• Proportion of stroke centers participating	• ADPH RESCUE ePCR
**Implementation**	• Proportion of stroke system patients with TCC-guided EMSA performed by EMS	• LifeTrac database
	• Proportion of EMS transports triaged past a non-MTC to a MTC when advised	• LifeTrac database
	• TCC and stroke center adherence to the region-specific triage plan	• LifeTrac and ADPH REDCap databases
	• Differential implementation for rural vs. urban populations	• LifeTrac and ADPH REDCap databases
**Maintenance**	• Sustained adoption and implementation over time	• LifeTrac and ADPH REDCap databases

*RE-AIM, Reach, Effectiveness, Adoption, Implementation, and Maintenance; TCC, Trauma Communications Center; SBST, Severity-Based Stroke Triage; ADPH, Alabama Department of Public Health; RESCUE ePCR, Recording of Emergency Medical Services Calls and Urgent-care Environment electronic Patient Care Reports; LVO, Large Vessel Occlusion; MT, Mechanical Thrombectomy; EMS, Emergency Medical Service; tPA, tissue Plasminogen Activator; MTC, Mechanical Thrombectomy Center*.

After implementation, we will use validated, quantitative surveys of stakeholders to assess perceptions of feasibility, appropriateness, and acceptability of the intervention (40). We will employ follow-up qualitative interviews with purposefully selected individuals to identify barriers and facilitators to adoption, implementation, maintenance, and spread using a mixed methods approach (AIM 3). This will help identify context-specific strategies to guide implementation efforts elsewhere.

### Data Management of Embedded Stepped Wedge Cluster Trial

**Figure 4:**
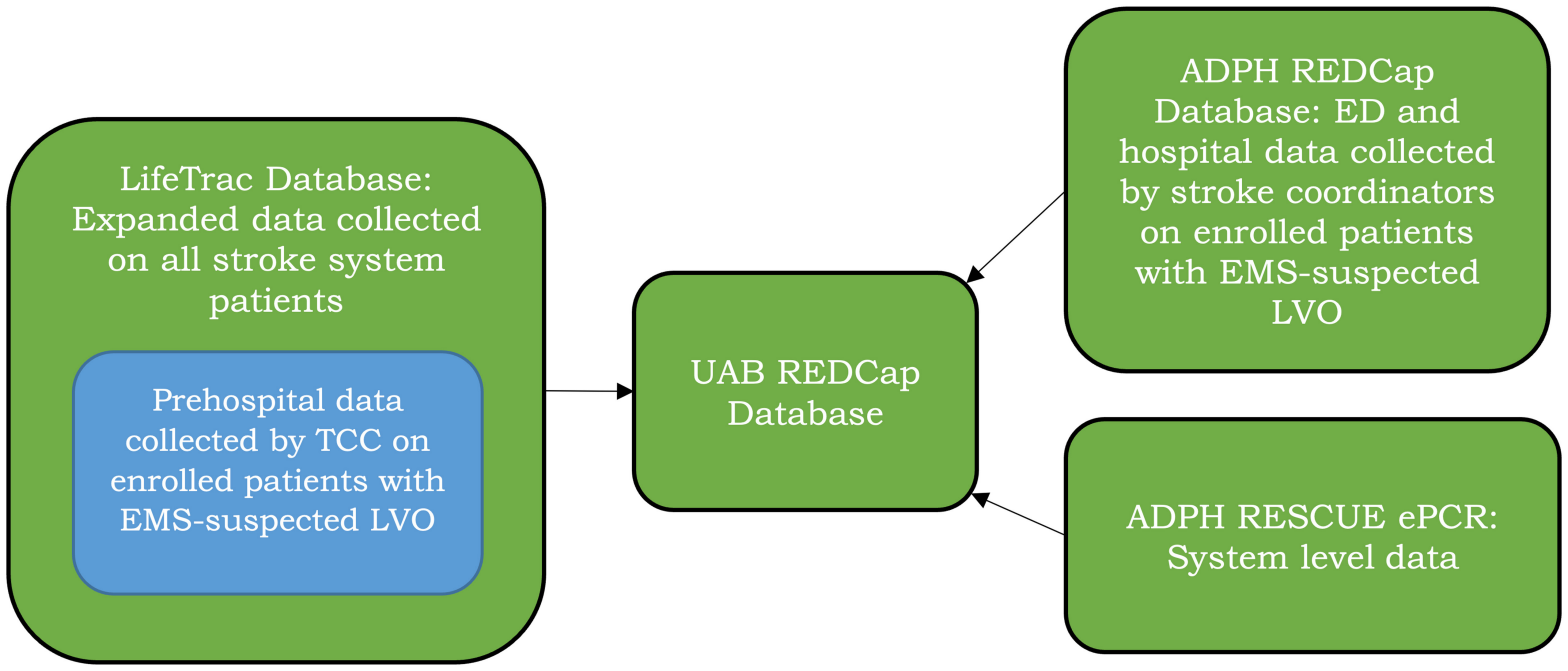
Data sources. (1) Expanded LifeTrac data entry on all stroke system patients with embedded LifeTrac data collection by TCC on study patients to both guide and document the prehospital triage process in real-time; (2) Study patient ED and hospital data entered by stroke coordinators into an ADPH REDCap database, and (3) System level data captured by ADPH's Recording of Emergency Medical Services Calls and Urgent-care Environment electronic Patient Care Reports (ADPH RESCUE ePCR).

Data sources are shown in Figure 4 and will include the following: (1) Expanded LifeTrac data entry by TCC on all stroke system patients to include prehospital EMSA items (collected in real-time by TCC communicators), abstracted hospital data collected by stroke coordinators including whether an LVO was confirmed, LVO location, and whether the patient received MT and embedded LifeTrac data collection by TCC on patients with EMS-suspected LVO who meet study entry criteria to both guide and document the prehospital triage process in real-time; (2) Study patient ED and hospital data entered by stroke coordinators into an ADPH REDCap database, and (3) System level data captured by ADPH's Recording of Emergency Medical Services Calls and Urgent-care Environment electronic Patient Care Reports to allow determination of the proportion of Alabama MT patients who undergo TCC Coordinated SBST, county and regional EMS response times, proportion of EMS organizations and stroke centers participating, and data on fidelity and maintenance of implementation. REDCap is a secure, HIPAA-compliant, web-based application for supporting data capture for research studies (41). The ADPH REDCap database was developed specifically for this study and will include applicable NINDS Common Data Elements from ED and hospital medical records including the National Institute of Health Stroke Scale (42, 43) and a structured telephone modified Rankin Scale (44, 45) at 3 months post-stroke.

Patients entered in the stroke system are assigned a unique TCC number that will enable tracking of the patient and linking of data. We plan to pilot data entry and tracking of patients through different phases (prehospital, ED, hospital) and venues (different EDs) of care by TCC and stroke center coordinators in two EMS regions prior to statewide stroke coordinator training. EMS Regional Agencies will play an important role in ensuring complete data capture from stroke centers by following up with stroke center coordinators. Encrypted ADPH data will be transferred to a UAB REDCap database for additional data cleaning, auditing, and analysis by dedicated study personnel including a data manager (MG) and project manager (SK). Data collection forms including validated stroke assessments are available (Supplementary Material 2). Data will be reviewed with the ADPH on a monthly basis. As this is an observational study of a change in health care policy by the ADPH, there is no data monitoring committee. As noted above, the State Medical Director of the OEMS (WC) will provide medical oversite for the OEMS as implementation goes statewide and provide updates to the Statewide Trauma and Health Systems Advisory Council.

### Sample Size Estimates for Stepped Wedge Cluster Trial

Based on 2018 data, the Alabama stroke system volume is about 7,630 over a 1-year period. Based on our pilot data (15), we estimate exclusion of 10% (763 patients) who are unresponsive or poorly responsive, and 5% (382 patients) with time last known well >24 h. We will allow for 10% missing EMSA data (763 patients), leaving 5,722 patients. Pilot data also indicate that over 1 year ~41% of this population (2,346 patients) will have EMSA ≥ 4 and be eligible for enrollment, 18% of enrolled patients (423 patients) will have LVO and be available for analysis of our primary endpoint, and ~50% of the patients with LVO are treated with MT (211 patients). Thus, for our power analyses, we will use 50% as the reference to examine whether implementation of TCC Coordinated SBST will be associated with a significant increase in the proportion of stroke system patients with suspected LVO who are treated with MT. Because of the Before-After nature of the data, a test that accounts for this dependency is necessary. However, even though these are repeated observations the dependency could be rather weak. Many of the same entities (e.g., hospitals, doctors, and paramedics) will be involve both before and after, the patients are very likely to be different; thus, leading to weaker dependency. To account for this possibility, we varied the strength of the dependency (correlation) from *r* = 0 to 0.5. Because the effect of implementing TCC Coordinated SBST is unknown, we varied the effect from small 10% to large 40% improvement. For a weak dependency of (*r* = 0.1), we would need ~350 subjects before and after to detect a moderate 20% improvement with 80% power at a α = 0.05 significance level. This corresponds to an increase of 35 patients treated with MT after TCC Coordinated SBST implementation. A smaller 15% improvement (increase of 26 patients treated with MT) with stronger dependency (*r* = 0.5) would also require ~350 subjects before and after to obtain 80% power at a α = 0.05 significance level. In either case, this data could be collected in ~20 months. Our sample size estimates are based on statewide stroke system entry volume data. Estimates of study eligibility volume and LVO frequency are based on methodology used in our preliminary study. However, to account for the possibility of smaller stroke system volume, smaller effect size, and/or weaker dependency, we plan on 36 months of data collection. For the proposed 36-month data collection timeline, with potentially 1,200–1,300 total observations, we should have more than adequate statistical power to detect a 15% improvement in the primary endpoint even with a weak dependency (*r* = 0.1) among observations.

### Patient Retention and Follow-Up

As noted above, we plan to pilot tracking of patients through different phases and venues of care to ensure that we retain all enrolled patients. Most of the patient data will be collected during the prehospital, ED, and hospital phases of care, so patient retention beyond the initial hospitalization is not expected to an issue for our primary endpoint. Collection of the follow-up modified Rankin Scale at 3 months (a secondary endpoint) will be facilitated by engagement of patients by local stroke coordinators at the time of their hospitalization.

### Mixed Methods Sample Estimates

Planned mixed methods data collection by stakeholder group including planned number of focus groups, surveys, and interviews is shown in Table 4. We will sample from each EMS region and adjust sampling strategy to account for the small number of stroke centers in EMS regions 2 and 4 to reach saturation (46) within and across groups. We plan to foster engagement of stakeholder groups in various ways to achieve adequate participation. Involvement of EMS will be facilitated by EMS regional directors and advisory councils, and stroke center coordinator and director participation will be promoted by regional educational conferences described above.

**Table 4:** Mixed methods data collection by stakeholder group.

**Stakeholder group**	**Stroke system role**	***N***	**Pre-implementation**	**Post-implementation**
**Alabama Trauma Communications Center**	Prehospital stroke care coordination	19	2 Focus groups (*n* = 19)	Survey (*n* = 19)
				Interviews (*n* = 12)
**EMS Regional Directors and Advisory Councils**	OEMS and EMS coordination	72	Individual interviews with Regional Directors (*n* = 6)	Regional Advisory Council Survey (*n* = 72)
				Interviews (*n* = 36)
**EMS**	Stroke assessment, care, triage	4,800	6 Focus groups (*n* = 48–72)	Survey (*n* = 600)
				Interviews (*n* = 36)
**Stroke Center Coordinators**	Stroke center care coordination	69	5 Focus groups (*n* = 40–60)	Survey (*n* = 69)
				Interviews (*n* = 30)
**Stroke Center Directors**	Stroke center leadership	69	Individual interviews (*n* = 30)	Survey (*n* = 69)
				Interviews (*n* = 30)

*EMS, Emergency Medical Service: OEMS, Office of Emergency Medical Services*.

### Statistical Analysis of Stepped Wedge Data

TCC Coordinated SBST will be implemented sequentially, thus leading to a stepped wedge design. For the primary endpoint, to account for the dependency among repeated observations over time, generalized linear mixed models and generalized estimating equations will be employed to model a binary outcome with a logit link function. However, we will explore other distribution possibilities (i.e., probit; log-binomial; negative binomial) to develop the best fitting model. For secondary endpoints that are categorical or ordinal (i.e., proportion treated with tPA; Rankin scores), we will use similar approaches. For secondary endpoints that are more continuous (e.g., time to tPA treatment), we will use linear mixed models assuming a normal distribution and Cox regression for survival analysis. Missing data will be analyzed by creating an indicator code for missingness to assess whether the missingness is systematic. If we conclude the data are missing at random then we will use multiple imputation and compare results with and without the imputation methodology. If we determine that the missingness is systematic, we will conduct sensitivity analyses to examine the extent the missing data have on the results, interpretations, and conclusions of the study.

### Interim Analysis of Stepped Wedge Data

The stepped wedge nature of the data allows opportunity for Interim Analyses after TCC Coordinated SBST is implemented in EMS Regions 3 and 5. Interim analysis of the primary endpoint will be used to guide power analysis for future data collection. Interim analysis on secondary endpoints will be used to optimize training and delivery of TCC Coordinated SBST for Regions 1, 2, 4, and 6.

### Qualitative and Mixed Methods Analysis

#### Qualitative Analysis

All interviews and focus groups will be transcribed verbatim using professional transcription services. Transcripts will be checked for accuracy and analyzed using NVivo® 12 Pro (QSR International, Burlington, MA). Qualitative analysis will be performed by experts under Dr. Ivankova's guidance. Transcripts will be analyzed using an inductive thematic approach (47). A constant comparative method will be used to generate and compare codes to other codes, codes to other categories, and categories to other categories (48). Several qualitative experts will independently code the data to minimize interpretation bias. Emergent codes and themes will be regularly discussed with the research team to jointly generate the code book to guide further analysis. Inter-coder agreement will be targeted at acceptable rate of 90% or higher (49). Content analysis (50) will be performed on the generated codes, categories and themes to quantitatively categorize the coded information based on the number of references made to a specific category or theme and to systematically represent consistencies in viewpoints across the stakeholders and sites. To ensure the validity of participants' responses and to emphasize the importance of their input, we plan to share the summaries of the interviews with participants and will seek additional clarifications and interpretation of their views on and perceptions of implementation (51).

#### Mixed Methods Analysis

In the pre-implementation phase, the quantitative and qualitative findings will be used to inform the implementation of TCC Coordinated SBST. In the post-implementation phase, the quantitative results from stakeholders' assessment of intervention's feasibility, appropriateness, and acceptability will inform the selection of participants for follow-up qualitative interviews to identify the perceived barriers and facilitators to adoption, implementation, maintenance, and spread of the intervention. The quantitative results will also provide a framework for developing the interview questions to further elaborate on the quantitative results (27). In the final stage, we will use integrative strategies such as developing joint displays (52) for side-by-side comparison of quantitative and qualitative results in summary tables (53) and figures (54) to fully understand and describe the contexts and processes for this intervention implementation.

### Study Timeline

**Figure 5:**
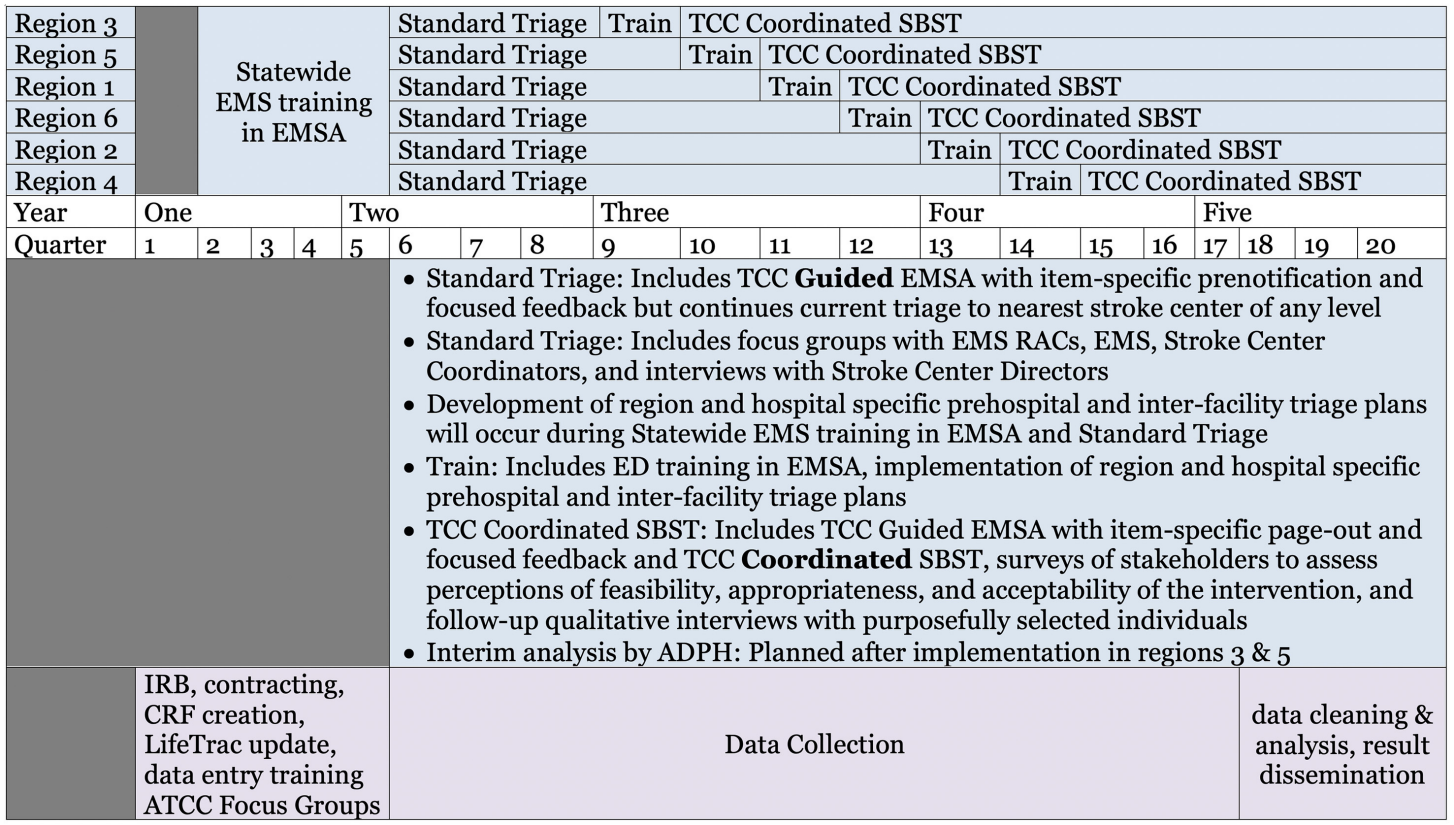
Five year timeline. Stepped wedge cluster trial with each EMS region serving as a cluster. During Standard Triage periods, we will implement TCC Guided EMSA but continue current triage to the nearest stroke center of any level and conduct focus groups and interviews to aid in the development of region and hospital specific SBST plans. During the Train periods, we will conduct regional educational symposia and implement SBST plans. During TCC Coordinated SBST periods TCC will guide EMS in performance of the EMSA and coordinate SBST and we will conduct stakeholder surveys and interviews to assess context-specific perceptions of the intervention.

The 5-year study timeline is shown in Figure 5. Based on the estimated sample size required to detect 15% improvement with 80% power, and given some uncertainty regarding effect size and dependency, we will allow for 36 months of patient data collection. At the time of this publication, focus groups are underway and patient enrollment is pending.

## Discussion

TCC Coordinated SBST aims to transform the acute stroke care system by coordinating prehospital and inter-facility emergency stroke care. This provides a “natural experiment” allowing assessment of both the public health impact and “how and why” of implementation of an innovative acute stroke care model based on Alabama's trauma system.

TCC Coordinated SBST differs significantly from the recently completed RACECAT trial. The RACECAT trial was well-designed and conducted but was unable to show the benefits of SBST for patients with suspected LVO (55, 56). The RACECAT trial compared two transport destinations (MTC vs. local non-MTC) for patients with suspected LVO in order to optimize access to MT, recognizing that direct transport to a MTC might delay or preclude tPA. Even though median time from onset to groin puncture was significantly shorter for LVO patients directly transported to MTCs (214 vs. 270 min, *p* < 0.001), direct transfer to a MTC did not lead to improved outcomes for LVO patients compared with initial transfer to a local non-MTC. It is not surprising that tPA was administered more frequently (60 vs. 48%, *p* < 0.001) and rapidly (onset to treatment 120 vs. 155 min, *p* < 0.001) at local centers compared to patients transported directly to MTCs. It may be that the benefits related to more rapid MT in patients transported directly to MTCs were balanced by the benefits of more frequent and rapid tPA treatment at non-MTCs.

As opposed to RACECAT, TCC Coordinated SBST will coordinate triage to optimize access to both tPA and MT. Thus, the current study hopes to avail patients with LVO the potential benefits of early bridging therapy with tPA at non-MTCs prior to MT. Two recent clinical trials comparing MT alone to MT following tPA have shown noninferiority of MT alone (57, 58). However, these studies evaluated bridging therapy administered at the mothership with median onset to tPA times of 184 and 166 min, respectively, and may not have captured the potential benefits of better thrombus resolution associated with shorter stroke onset to tPA administration or longer dwell times of tPA (59). Interestingly, one recent RCT that failed to demonstrate noninferiority with regard to functional outcome after MT alone had a shorter mean onset to tPA treatment time of 150 min (60). Studies of LVO patients transferred for MT have documented the association of tPA with increased rates of recanalization prior to MT (61, 62), and the need for fewer passes during MT to achieve successful recanalization (63). A very recent meta-analysis concluded that compared to MT alone, bridging therapy led to better clinical outcomes, lower mortality at 90 days, and higher successful recanalization rates, without increasing the risk of near-term hemorrhagic complications (64).

Beyond potential benefits of tPA in patients with LVO, it is important to recognize that prehospital severity-based stroke triage is imperfect and that existing severity scales are subject to false negatives in patients with LVO and milder stroke, as well as false positives in patients with non-LVO stroke, stroke mimics or hemorrhagic strokes (65, 66). In RACECAT, a RACE of ≥ 5 resulted in ~33% of false transfers for MT (56). TCC Coordinated SBST hopes to facilitate access to tPA for all patients with ischemic stroke who are candidates for this therapy regardless of LVO status.

In the emphasis on access to both tPA and MT, TCC Coordinated SBST is similar to the American Heart Association's Mission: Lifeline Stroke Severity-Based Stroke Triage Algorithm for EMS (9). If successful, TCC Coordinated SBST will provide validation of a model of SBST. However, the algorithm does not fully address the fragmentation of our acute stroke care system since it does not address the issue of EMS medical control, ED care, or interfacility transfer for patients initially transported to a non-MTC. The potential to reduce the time from stroke patient arrival at a non-MTC to thrombectomy was demonstrated in a study through a standardized process consisting of early MTC notification, CT angiography at the non-MTC, and electronic image sharing prior to transfer (67). An important aspect of the current protocol is continued coordination of ED care and inter-facility transfer if needed for patients initially triaged to a non-MTC. In this regard we hope to emulate the success of RACECAT in achieving door-in door-out times of ≤ 60 min (56).

Part of the innovation of this project is related to the hybrid type 1 effectiveness-implementation design (68) and a mixed methods methodological approach (24) to guide the development of this intervention and fully understand the contexts and processes for its implementation. This approach is appropriate: we have ample evidence to support the efficacy of MT in the treatment of LVO, but lack an integrated, readily adoptable, implementable, and sustainable approach to optimize timely access to MT. Further, this is a complex intervention, depending on the intervention itself and the context in which it is placed (28). Accordingly, we will collaborate with stakeholders to refine the context specific forms needed to achieve the core functions of the program (30). Yet, while adaptation of this stroke system change to the Alabama stroke system is fundamental to the success of the initiative, we hope that the process will lend itself to implementation in other contexts. An innovative aspect of this project is that it leverages existing trauma system infrastructure as the basis for a more integrated and effective system of emergency stroke care. The coordinating function in this study will be carried out by experienced paramedics who serve as Trauma Communications Center communicators. This may increase the ability to generalize our findings to other regions and states.

## Ethics and Dissemination

The Principal Investigator (TIG) will have overall responsibility for study design, integrity of data collection and analysis, communicating important protocol modifications to relevant parties, and dissemination of project findings including report and article writing. Patients will receive standard ED and hospital evaluation and treatment as clinically indicated. The main risk of participating in the study is the loss of confidentiality of medical information. The investigators will take every precaution to protect privacy through use of best practices for data analysis and storage, limiting access to information identifying individuals, and properly disposing of any materials which are not needed and may disclose subject information. The protocol, and patient Waivers of HIPAA and Informed Consent were approved by the UAB and ADPH Institutional Review Boards. As with patients, the investigators will take every precaution to protect privacy of stakeholder participants. The TCC focus group protocol, Waiver of HIPAA, and Waiver of Consent Documentation were approved by the UAB Institutional Review Board. IRB approvals will be obtained prior to undertaking planned mixed methods study procedures. The focus group or interview protocol will be explained to stakeholder participants by SK or TIG. An Information Sheet will be provided to all participants. An example Information Sheet is available (Supplementary Material 3). The study protocol (original) was registered on July 27, 2021 at ClinicalTrials.gov (https://clinicaltrials.gov), and the unique identifier number is NCT04978480.

## Ethics Statement

The protocol and patient Waivers of HIPAA and Informed Consent were approved by the University of Alabama at Birmingham Institutional Review Board [Federalwide Assurance # FWA00005960, IORG Registration # IRB00000196 (IRB 01), IORG Registration # IRB00000726 (IRB 02), and IORG Registration # IRB00012550 (IRB 03)] on May 18, 2020, and by the Alabama Department of Public Health Department Overview and Approval of Research Committee on November 9, 2020. The ATCC focus group protocol, Waiver of HIPAA, and Waiver of Consent Documentation were approved by the University of Alabama at Birmingham Institutional Review Board on February 24, 2021. IRB approvals will be obtained prior to undertaking planned mixed methods study procedures. Written informed consent for participation was not required for this study in accordance with the national legislation and the institutional requirements.

## Author Contributions

TG conceived and designed the work and drafted the manuscript. NI made substantial contributions to the conception and design of the mixed methods approach and was a major contributor in writing the manuscript. MB made substantial contributions to the conception and design of the stepped wedge cluster trial and was a major contributor in writing the manuscript. EH made substantial contributions to the conception and design of outcomes related to public health and stakeholder perceptions and was a major contributor in writing the manuscript. BM made substantial contributions to the conception and design of the implementation science approach and was a major contributor in writing the manuscript. MG made substantial contributions to the design of the work and created the REDCap database. MM made substantial contributions to the design of the work and LifeTrac upgrades. WC, AF, GV, ML, CS, JO, KW, JG, and SK made substantial contributions to the design of the work. All authors read and approved the final manuscript (69).

## Funding

The project was supported by the National Institute of Neurological Disorders and Stroke under award number R01NS117813. The funding agency played no role in the design of the study and collection, analysis, and interpretation of data and in writing the manuscript.

## Conflict of Interest

The authors declare that the research was conducted in the absence of any commercial or financial relationships that could be construed as a potential conflict of interest.

## Publisher's Note

All claims expressed in this article are solely those of the authors and do not necessarily represent those of their affiliated organizations, or those of the publisher, the editors and the reviewers. Any product that may be evaluated in this article, or claim that may be made by its manufacturer, is not guaranteed or endorsed by the publisher.

## Abbreviations

ADPH: Alabama Department of Public Health
CT: Computerized Tomography
TCC: Trauma Communications Center
ED: Emergency Department
EMS: Emergency Medical Service
EMSA: Emergency Medical Stroke Assessment
LVO: Large Vessel Occlusion
MT: Mechanical Thrombectomy
MTC: Mechanical Thrombectomy Center
OEMS: Office of Emergency Medical Services
SBST: Severity-Based Stroke Triage
RE-AIM: Reach, Effectiveness, Adoption, Implementation and Maintenance
tPA: tissue Plasminogen Activator.
