# Supplementary material for: Trauma Communications Center Coordinated Severity-Based Stroke Triage: Protocol of a Hybrid Type 1 Effectiveness-Implementation Study
Source: Front Neurol. 2021 Dec 6;12:788273. doi: 10.3389/fneur.2021.788273 (PMC8686821; doi:10.3389/fneur.2021.788273)
Supplement: Supplementary file 1 [file Data_Sheet_1.PDF]

Focus Group Guide (ATCC)

Good evening and thank you for joining us today. My name is_____and assisting me is____.

You were invited to this focus group because we are interested in identifying potential barriers and facilitators to prehospital identification of stroke in general, severe stroke (suspected large-vessel occlusion), entry of patients into the Alabama stroke system, and coordination of prehospital and inter-facility emergency stroke care by TCC.

This will be an informal discussion and we want you to share your honest opinions. We would like for you to share your thoughts and opinions even if they are different from everyone else's. There are no right or wrong answers. All thoughts are important. We do not want to miss your comments; therefore, the session will be recorded. This discussion will last about 1.5 hours and you will receive a $10 gift card for your participation.

Review ground rules

We have several questions to go through and we want to try to let everyone answer them in approximately 1.5 hours. Let's follow some guidelines in order to make the best use of our time:
- Please speak up so everybody can hear.
- Please speak one at a time.
- Please avoid cell phone distractions/ringing.
- Please do not share what is said during the session with others outside the group. This conversation is confidential.
- We will not take a formal break, but feel free to step away for a minute or two if needed.

Now, we are ready for the discussion and will turn on the recorder.

Let's begin

Q. 1.

How familiar are you with treatment options for acute ischemic stroke?

Probes:

- What do you know about the time windows for those treatments?
- What training have you received about stroke?
- How effective was this training in improving your stroke knowledge?
- How effective was this training in improving your stroke assessment skills?
- How comfortable are you with the role that you will have in severity-based stroke triage?
- How comfortable do you think EMS providers will be with the role that you will have in severity-based stroke triage?

Q. 2.

What are some factors that make EMS stroke recognition difficult?

Probes:

- Why do you think stroke recognition can be challenging?
- How would you help EMS providers assess patients who are unable to follow commands or have a language barrier?
- How do EMS providers make a decision to enter suspected stroke patients in the stroke system?
    - Are all suspected stroke patients entered in the stroke system? If not, why?

Q. 3.

What are some factors that you think can facilitate stroke recognition and/or stroke system entry?

Probes:

- Why do you think it/these are important?
- How can it/these be implemented?
- What do you think you need to implement it/these?

Q. 4.

What EMS protocol driven assessments do you use to identify stroke severity?

Probes:

- How do you use stroke scales?
    - Are they a required part of the evaluation? Why or why not?
    - Do they indicate severity of stroke? Please explain.
- How comfortable are you with diagnosis of suspected LVO on the basis of a stroke scale?
    - Do you think if EMS should report EMSA items to ATCC without need for guidance? Why or why not?
    - Do you think if ATCC should guide EMS in performance of EMSA items? Why or why not?
    - How comfortable do you think EMS providers will be with diagnosis of suspected LVO on the basis of a stroke scale?
- Do you think we should focus on left and right MCA syndromes instead of the diversity of stroke presentations? Why or why not?
- What strategies do you think would help better detect neglect if it was fast and accurate?
- What do you think about EMS use of an ancillary test (such as a POCT) for stroke or LVO?
    - How acceptable would it be to EMS providers?

Q. 5.

What is the process of routing patients who have been entered into the stroke system?

Probes:

- How does ATCC assist in choice of destination/coordination of transport mode?
- Are there stroke centers you regularly bypass? If so, why?
- Are patients with suspected stroke routed differently on the basis of perceived severity? Please explain.
- How comfortable are you with the idea of advising EMS providers to bypass a level 2 or 3 stroke

center to transport a patient with suspected LVO to a thrombectomy capable hospital?

- Should there be a standard transport time limit to a thrombectomy capable hospital for patients with suspected LVO? Why or why not?
    - What do you think a transport time limit should be?
- What feedback do you receive from the hospital on stroke patient status?
    - How do you use this feedback?

Q. 6.

What is the process of transferring patients with confirmed LVO from a non-thrombectomy capable to a thrombectomy capable hospital?

Probes:

- How comfortable are you following up with level 2 and 3 stroke centers every 20 minutes to check on status of stroke system patients with suspected LVO?
- How comfortable are you assisting level 2 and 3 stroke centers with the process of transfer to a thrombectomy capable hospital for patients who are candidates for mechanical thrombectomy?
- How early should we mobilize a transport team for transfer of a suspected LVO patient from a non-thrombectomy capable to a thrombectomy capable hospital ("expedited transport process")?
    - At the time the initial routing decision to a non-thrombectomy capable hospital is made (that is, prior to arrival at the initial hospital destination)?
    - After the initial ED evaluation and EMSA at the non-thrombectomy capable hospital?
    - After the head CT scan at the non-thrombectomy capable hospital?
- How likely do think that an expedited transport process would happen? What would bet the barriers?
- Do you think that an EMS unit that transports a suspected LVO patient to a level 2 or 3 Stroke Center should remain until a transfer decision is made? Please explain.
- Do you think that patients with confirmed LVO at a level 2 or 3 stroke center should be transported directly to the thrombectomy capable hospital angiography suite? Please explain.

Q. 7.

Do you think the stroke emergency medical system should be modelled after the trauma system? Why or why not?

Thank you all for participating in this discussion today! The focus group is adjourned.
