# Supplementary material for: Trauma Communications Center Coordinated Severity-Based Stroke Triage: Protocol of a Hybrid Type 1 Effectiveness-Implementation Study
Source: Front Neurol. 2021 Dec 6;12:788273. doi: 10.3389/fneur.2021.788273 (PMC8686821; doi:10.3389/fneur.2021.788273)
Supplement: Supplementary file 2 [file Data_Sheet_2.PDF]

Confidential

Emergency Medical Services

Study ID	________________
TCC number	________________
EMS provider	________________
Date of initial call	________________ (MM/DD/YYYY)
Time of initial call	________________
Date of arrival at scene	________________ (MM/DD/YYYY)
Time of arrival at scene	________________
County of initial contact	________________
ZIP Code	________________
Age	________________
Sex	◯ Female<br>◯ Male<br>◯ Unknown/Not reported
Was the time of patient's last known well noted?	◯ Yes<br>◯ No
Date of last known well	________________
Time of last known well	________________
Alert Voice Pain Unresponsive (AVPU) assessment	◯ Alert and oriented<br>◯ Verbally Stimulated<br>◯ Pain Stimulated<br>◯ Unconscious
Systolic Blood Pressure Reading (mmHg)	________________

Diastolic Blood Pressure Reading (mmHg)	__________________________________
Glucometer reading (mg/dL)	__________________________________
Glascow Coma Scale	__________________________________
EMSA: Horizontal gaze	○ Normal<br>○ Abnormal
EMSA: Facial weakness	○ Normal<br>○ Abnormal
EMSA: Arm weakness	○ Normal<br>○ Abnormal
EMSA: Leg weakness	○ Normal<br>○ Abnormal
EMSA: Naming	○ Normal<br>○ Abnormal
EMSA: Repition	○ Normal<br>○ Abnormal
Is the patient an tPA candidate?	○ Yes<br>○ No
Estimated transport time to nearest stroke center	__________________________________
Date of departure from scene	__________________________________<br>(MM/DD/YYYY)
Time of departure from scene	__________________________________
Mode of Transportation	__________________________________
Hospital selection	__________________________________
Hospital patient was transported to	__________________________________
Has severity based stroke triage been activated in the EMS region?	○ Yes<br>○ No

Triage Activation

Study ID	__________________________
Transport past a non-MTC to a MTC (bypass) advised?	◯ Yes<br>◯ No
Bypass to MTC accepted?	◯ Yes<br>◯ No
Reason why bypass to a MTC was not accepted?	◯ Patient/family request<br>◯ EMS decision<br>◯ patient's clinical condition warrants transport to closer facility<br>◯ Other
Other reason why bypass to a MTC was not accepted?	__________________________________
Why wasn't bypass to a MTC advised?	◯ transport to MTC will preclude use of tPA<br>◯ Additional time for transport to MTC exceeds region-specific transport time limits<br>◯ patient's clinical condition warrants transport to closer facility<br>◯ NA (nearest stroke center is a mtc)<br>◯ other
Other reason bypass to a MTC was not advised?	__________________________________
Transport to a non-MTC accepted?	◯ Yes<br>◯ No
Reason why transport to a non-MTC was not accepted?	◯ Patient/family request<br>◯ EMS decision<br>◯ Other
Other reason why transport to a non-MTC was not accepted?	__________________________________

Emergency Department 1

Study ID	__________________
Stroke Center Name	__________________
Date of arrival to ED	__________________ (MM/DD/YYYY)
Time of arrival to ED	__________________
Was the patient transferred from another hospital?	◯ Yes<br>◯ No
Hospital the patient has been transferred from	__________________
Was the patient transferred to another hospital?	◯ Yes<br>◯ No
Hospital the patient was transferred to	__________________
Date of last known well	__________________ (MM/DD/YYYY)
Time of last known well	__________________
Was EMSA done in the ED?	◯ Yes<br>◯ No
EMSA: Horizontal gaze	◯ Normal<br>◯ Abnormal
EMSA: Facial weakness	◯ Normal<br>◯ Abnormal
EMSA: Arm weakness	◯ Normal<br>◯ Abnormal
EMSA: Leg weakness	◯ Normal<br>◯ Abnormal
EMSA: Naming	◯ Normal<br>◯ Abnormal
EMSA: Repition	◯ Normal<br>◯ Abnormal

Was NIHSS done in the ED?	◯ Yes<br>◯ No
Level of consciousness	◯ 0 Alert<br>◯ 1 drowsy<br>◯ 2 stupor<br>◯ 3 comatose
Level of consciousness questions	◯ 0 both<br>◯ 1 one<br>◯ 2 neither
Level of consciousness commands	◯ 0 both<br>◯ 1 one<br>◯ 2 neither
Best gaze	◯ 0 Normal<br>◯ 1 Partial gaze palsy<br>◯ 2 Forced deviation
Visual Fields	◯ 0 No visual loss<br>◯ 1 Partial hemianopia<br>◯ 2 Complete hemianopia<br>◯ 3 Bilateral hemianopia
Facial Palsy	◯ 0 Normal<br>◯ 1 Minor paralysis<br>◯ 2 Partial paralysis<br>◯ 3 Complete paralysis
Motor arm, Left arm	◯ 0 No drift<br>◯ 1 Drift<br>◯ 2 Some effort against gravity<br>◯ 3 No effort against gravity<br>◯ 4 No movement<br>◯ UN: amputation or joint fusion
Motor arm, Right arm	◯ 0 No drift<br>◯ 1 Drift<br>◯ 2 Some effort against gravity<br>◯ 3 No effort against gravity<br>◯ 4 No movement<br>◯ UN: amputation or joint fusion
Motor leg, Left leg	◯ 0 No drift<br>◯ 1 Drift<br>◯ 2 Some effort against gravity<br>◯ 3 No effort against gravity<br>◯ 4 No movement<br>◯ UN: amputation or joint fusion
Motor leg, Right leg	◯ 0 No drift<br>◯ 1 Drift<br>◯ 2 Some effort against gravity<br>◯ 3 No effort against gravity<br>◯ 4 No movement<br>◯ UN: amputation or joint fusion

Limb Ataxia	◯ 0 Absent<br>◯ 1 Present in one limb<br>◯ 2 Present in two limbs<br>◯ UN: Amputation or joint fusion
Sensory	◯ 0 Normal<br>◯ 1 Mild to moderate sensory loss<br>◯ 2 Severe or total sensory loss
Best Language	◯ 0 No Aphasia<br>◯ 1 Mild to moderate aphasia<br>◯ 2 Severe aphasia<br>◯ 3 Mute, global aphasia
Dysarthria	◯ 0 Normal<br>◯ 1 Mild to Moderate Dysarthria<br>◯ 2 Severe dysarthria<br>◯ UN: Intubated or other physical barrier
Extinction and Inattention	◯ 0 No abnormality<br>◯ 1 partial<br>◯ 2 complete
Initial NIH stroke scale, total	__________________
Date of CT	__________________<br>(MM/DD/YYYY)
Time of CT	__________________
Diagnosis of ischemic stroke causing measurable neurological deficit or disability?	◯ Yes<br>◯ No
Was patient given tPA?	◯ Yes<br>◯ No
Reason patient was not given tPA	◯ Patient is outside the tPA treatment window<br>◯ Uncontrolled hypertension at time of treatment (Blood pressure > 185/110)<br>◯ Evidence of intracranial hemorrhage on baseline CT scan<br>◯ CT demonstrates multilobar infarction (hypodensity >1/3 cerebral hemisphere)<br>◯ Patient is anticoagulated (warfarin, heparin, LMWH, or DOACs)<br>◯ Other
Other reason patient was not given tPA	__________________
Date of tPA initiation	__________________<br>(MM/DD/YYYY)

Time of tPA initiation	__________________________________
Is LVO present?	◯ Yes<br>◯ No
How was LVO determined?	◯ CTA<br>◯ MRA<br>◯ cerebral angiogram
Date of LVO imaging	__________________________________<br>(MM/DD/YYYY)
Time of LVO imaging	__________________________________
Where is LVO?	◯ ICA<br>◯ MCA stem (M1)<br>◯ MCA division (M2)<br>◯ basilar artery<br>◯ other
Other LVO site?	__________________________________
Did patient get MT?	◯ Yes<br>◯ No
Reason patient did not get MT	◯ Extensive CT changes (low ASPECTS)<br>◯ ischemic core too large on perfusion imaging<br>◯ no significant perfusion mismatch<br>◯ Patient is outside MT treatment window<br>◯ Neurological deficit too mild<br>◯ other
Other reason patient did not get MT	__________________________________
Date of MT groin puncture	__________________________________<br>(MM/DD/YYYY)
Time of MT groin puncture	__________________________________
TICI grade post reperfusion treatment	__________________________________

Hospital Data

Study ID	__________________________________
Stroke Center Name	__________________________________
Date of Birth	__________________________________
Age	__________________________________
Sex	◯ Female<br>◯ Male<br>◯ Unknown/Not reported
Ethnicity	◯ Hispanic or Latino<br>◯ Not Hispanic or Latino<br>◯ Unknown / Not Reported
Race	◯ American Indian/Alaska Native<br>◯ Asian<br>◯ Native Hawaiian or Other Pacific Islander<br>◯ Black or African American<br>◯ White<br>◯ More Than One Race<br>◯ Unknown / Not Reported
ZIP Code	__________________________________
History of hypertension	◯ Yes<br>◯ No
History of diabetes mellitus	◯ Yes<br>◯ No
History of hyperlipidemia	◯ Yes<br>◯ No
History of coronary artery disease	◯ Yes<br>◯ No
History of congestive heart failure	◯ Yes<br>◯ No
History of chronic kidney failure	◯ Yes<br>◯ No
Acute kidney injury	◯ Yes<br>◯ No

History of atrial fibrillation	◯ Yes<br>◯ No
Previous stroke	◯ Yes<br>◯ No
Previous TIA	◯ Yes<br>◯ No
Current or previous tobacco use	◯ Yes<br>◯ No
Current or previous alcohol abuse	◯ Yes<br>◯ No
Baseline modified rankin score	__________________________________
Date of discharge	__________________________________
Discharge Diagnosis	◯ Ischemic stroke<br>◯ Intracerebral hemorrhage<br>◯ Transient Ischemic Attack<br>◯ Subarachnoid Hemorrage<br>◯ Non stroke related diagnosis
Discharge disposition	◯ Home<br>◯ Rehabilitation Facility<br>◯ Specialized Nursing Facility<br>◯ Long Term Assisted Care Facility or other hospital<br>◯ Other<br>◯ Deceased
Discharge modified rankin score	__________________________________
Date of 90 day modified rankin score (14 days)	__________________________________
90 day modified rankin score	__________________________________
