# Supplementary material for: Trauma Communications Center Coordinated Severity-Based Stroke Triage: Protocol of a Hybrid Type 1 Effectiveness-Implementation Study
Source: Front Neurol. 2021 Dec 6;12:788273. doi: 10.3389/fneur.2021.788273 (PMC8686821; doi:10.3389/fneur.2021.788273)
Supplement: Supplementary file 3 [file Data_Sheet_3.PDF]

INFORMATION SHEET

Title of Research: Trauma Communications Center Coordinated Severity-Based Stroke Triage

UAB IRB Protocol #: IRB-300005312

Principal Investigator: Toby I. Gropen, MD

Sponsor: National Institute of Neurological Disorder and Stroke

You are being asked to take part in either a focus group or interview as part of a research study because of your area of professional expertise. This study is being carried out by the University of Alabama at Birmingham (UAB) in collaboration with the Alabama Department of Public Health (ADPH). The overall goal is to study the implementation of a new model of severity-based stroke triage by ADPH modelled after Alabama's successful trauma system. We are utilizing focus groups and interviews to better understand the feasibility, appropriateness, and acceptability of this model and to identify and potential barriers to the implementation.

The objectives of the study are to-

- compare the proportion of patients encountered by the emergency medical service (EMS) with suspected large vessel occlusion (LVO) before and after implementation of TCC coordinated SBST;
- assess the public health impact of TCC coordinated SBST by using the RE-AIM framework; and
- assess stakeholder perceptions of the intervention's feasibility, appropriateness, and acceptability and identify the barriers.

This project, if successful, can serve as a model for how the trauma system infrastructure that already exists in other regions and states can serve as the basis for a more integrated and effective system of emergency stroke care.

We will enroll approximately 342 individuals to participate in focus groups or interviews. Focus groups or interviews will take place virtually (via zoom) that will last up to 1.5 hours. The focus group or interview will include a discussion asking you to share your opinions about the stroke system triage in Alabama. You will also complete a brief questionnaire about yourself and your professional experience.
Focus groups or interviews will be audio/video recorded and analyzed for research purposes. The audio recording may be sent to a transcription service, however, only your first name will be used during the focus group/ interview.

You will be paid $10 within 30 days of your focus group or interview. Ask the interviewer about the method of payment that will be used for this study (e.g., check, gift card).

Your participation in this research is strictly voluntary, and you can end your participation in the focus group or interview at any time. Although I will know your identity and contact information, I will keep this information separate from your interview responses and will destroy this information as soon

as it is no longer needed. Your data will be kept confidential; however, organizations involved in the project directly (i.e., ADPH, the UAB Department of Neurology) or those organizations involved the oversight of research (i.e., the UAB Institutional Review Board, The Office for Human Research Protections) may have access to identifiable information about you.

Some of the questions we ask may not be comfortable for you to answer when sharing in a group setting. You can choose not to answer any questions.

You will not directly benefit from participating. We hope the information learned from this study will serve as the basis for a more integrated and effective system of emergency stroke care.

Who may use and give out information about you?
Information about you may be used and given to others by the study doctor and staff. They might see the research information during and after the study.

Who might get this information?
All individuals/entities listed in the informed consent document(s), including but not limited to, the physicians, nurses and staff and others performing services related to the research (whether at UAB or elsewhere). Your information may also be given to the sponsor of this research. "Sponsor" includes any persons or companies that are working for or with the sponsor, or are owned by the sponsor, or are providing support to the sponsor (e.g., contract research organization).

Information about you and your health which might identify you may be given to:

- the Office for Human Research Protections (OHRP)
- the U.S. Food and Drug Administration (FDA)
- Department of Health and Human Services (DHHS) agencies
- Governmental agencies in other countries
- Governmental agencies to whom certain diseases (reportable diseases) must be reported
- ADPH

Why will this information be used and/or given to others?
Information about you that might identify you may be given to others to carry out the research study. The sponsor will analyze and evaluate the results of the study. In addition, people from the sponsor and its consultants will be visiting the research site. They will follow how the study is done, and they will be reviewing your information for this purpose.

May I withdraw or revoke (cancel) my permission?
Yes, but this permission will not stop automatically. The use of your information will continue until you cancel your permission.

You may withdraw or take away your permission to use and disclose your information at any time. You do this by sending written notice to the study doctor. If you withdraw your permission, you will not be able to continue being in this study.

When you withdraw your permission, no new information which might identify you will be gathered after that date. Information that has already been gathered may still be used and given to others. This would be done if it were necessary for the research to be reliable.

If you have any questions, concerns, or complaints about the research please contact the Principal Investigator, *Dr. Gropen,* at (205) 975-8569. If you have questions about your rights as a research participant, or concerns or complaints about the research, you may contact the UAB Office of the IRB (OIRB) at (205) 934-3789 or toll free at 1-855-860-3789. Regular hours for the OIRB are 8:00 a.m. to 5:00 p.m. CT, Monday through Friday.
